# Impacts of Psychological Stress on Osteoporosis: Clinical Implications and Treatment Interactions

**DOI:** 10.3389/fpsyt.2019.00200

**Published:** 2019-04-09

**Authors:** Ryan R. Kelly, Lindsay T. McDonald, Nathaniel R. Jensen, Sara J. Sidles, Amanda C. LaRue

**Affiliations:** ^1^Research Services, Ralph H. Johnson VA Medical Center, Charleston, SC, United States; ^2^Department of Pathology and Laboratory Medicine, Medical University of South Carolina, Charleston, SC, United States

**Keywords:** osteoporosis, bone, psychological stress, mental health, depression, PTSD, pharmacology, alternatives

## Abstract

The significant biochemical and physiological effects of psychological stress are beginning to be recognized as exacerbating common diseases, including osteoporosis. This review discusses the current evidence for psychological stress-associated mental health disorders as risk factors for osteoporosis, the mechanisms that may link these conditions, and potential implications for treatment. Traditional, alternative, and adjunctive therapies are discussed. This review is not intended to provide therapeutic recommendations, but, rather, the goal of this review is to delineate potential interactions of psychological stress and osteoporosis and to highlight potential multi-system implications of pharmacological interventions. Review of the current literature identifies several potentially overlapping mechanistic pathways that may be of interest (e.g., glucocorticoid signaling, insulin-like growth factor signaling, serotonin signaling) for further basic and clinical research. Current literature also supports the potential for cross-effects of therapeutics for osteoporosis and mental health disorders. While studies examining a direct link between osteoporosis and chronic psychological stress are limited, the studies reviewed herein suggest that a multi-factorial, personalized approach should be considered for improved patient outcomes in populations experiencing psychological stress, particularly those at high-risk for development of osteoporosis.

## Background

Emerging evidence points to the potential pathological impact of mental health on disease. It has long been held that stress has negative impacts on health and disease risk, but the specific mechanisms by which this occurs, as well as implications for treatments and clinical recommendations, have not been examined in-depth. This review will provide an overview of recent literature regarding the impact of psychological stress and stress-related disorders, such as post-traumatic stress disorder (PTSD), depression, and anxiety, on risk and treatment of osteoporosis. In this review, we first highlight mechanisms that impact both bone health and mental health toward identification of potentially overlapping signaling pathways. We then review current literature regarding the impact of common therapeutic agents for treatment of osteoporosis and mental health disorders. This will promote recognition of the potential interaction of these therapeutic agents in patients with concurrent mental health disorders and osteoporosis to encourage a broad view of disease management toward improved patient health. Finally, we provide a perspective outlook on the potentially beneficial effects of alternative treatments, such as exercise and nutritional supplementation, on both osteoporosis and psychological stress.

## Osteoporosis

There are four major bone cell types: osteoclasts, osteoblasts, osteocytes, and osteogenic stem cells. Osteoclasts, which are of myeloid origin, are giant, multinucleated cells that adhere to the bone and resorb it through acidification and proteolytic digestion. Osteoblasts counteract osteoclast-mediated bone resorption by secreting osteoid, which mineralizes to form new bone. There is a tightly-regulated balance between osteoclast-mediated removal of old or damaged bone and osteoblast-mediated replacement of new bone to maintain bone mass and skeletal homeostasis. After secretion of osteoid, osteoblasts either become trapped within the osteoid and terminally differentiate into osteocytes, quiesce into bone lining cells, or undergo apoptosis. Osteocytes comprise 90–95% of the bone cell population (1). Once they become embedded in the mineralized tissue, they develop cytoplasmic projections that intercalate throughout the bone, creating a signaling network to communicate directly with other osteocytes (2). Through this network, osteocytes regulate phosphate homeostasis and transduce mechanical stress signals into biologic activity to stimulate either bone resorption or formation. Osteogenic stem cells are the source of osteoblasts and osteocytes and are involved in bone repair, regeneration, and development. The functions and number of these cell types can become disrupted following bone damage or in disease states, such as osteoporosis.

Osteoporosis is the most common form of metabolic bone disease and is characterized by low bone mass and micro-architectural bone deterioration. The World Health Organization defines osteoporosis as a bone mineral density (BMD) that is ≤2.5 standard deviations below peak bone mass, which is typically achieved around age 30. In the United States alone, osteoporosis accounts for over 1.5 million fractures per year (3). By 2025, treatment costs are estimated to exceed $25 billion (4). Osteoporosis is characterized by an imbalance of skeletal remodeling, resulting in increased osteoclast activity and/or decreased numbers of osteoblasts, which can lead to decreased bone strength and mass, as well as increased susceptibility to fracture.

Osteoporosis is an umbrella term for a group of distinct pathological conditions and has been traditionally classified into primary and secondary types based on mechanism of disease (5). There are two main types of primary osteoporosis: type I osteoporosis and type II osteoporosis. Type I osteoporosis occurs most frequently in postmenopausal women and primarily results from estrogen deficiency. Estrogens inhibit production of receptor activator of nuclear factor kappa-B ligand (RANKL), which is crucial for osteoclast differentiation and recruitment, as well as interleukin (IL)-1, IL-6, and tumor necrosis factor-alpha (TNF-α) (6, 7). In addition, estrogens promote osteoblast differentiation and positively regulate several anabolic bone-related proteins, including insulin-like growth factor-1 (IGF-1), bone morphogenetic proteins (BMPs), and procollagen type I (COL1) (8). Thus, postmenopausal decrease in estrogen may affect both bone resorption and bone formation. The functional outcome is a rate of bone resorption that is higher than that of bone formation, resulting in a net decrease in bone mass. Type II osteoporosis is associated with aging and is commonly observed in men and women after the age of 60. Aging results in a progressive decline in osteoblast numbers and decreased osteoblast activity, but no change in osteoclast activity. It is still unknown how the cellular and molecular mechanisms that contribute to these two primary types of osteoporosis compare to each other or to what extent sex steroid deficiency contributes to age-related skeletal degradation. Findings in mouse models suggest that the effects of age on skeletal health are independent of estrogens, but data describing a similar mechanism in humans is lacking (9, 10).

Secondary osteoporosis is characterized by bone loss resulting from an underlying etiology, such as Cushing's syndrome, or prolonged treatment with glucocorticoids. In glucocorticoid-induced osteoporosis, bone loss occurs within several months of glucocorticoid treatment and can lead to significant decreases in cancellous bone mass and increased fracture risk. Excess glucocorticoids exert an inhibitory effect on osteoblast differentiation (11). Glucocorticoid-induced osteoporosis is the most common form of secondary osteoporosis and is the most common form of osteoporosis among young people [reviewed in Briot and Roux (12)]. Secondary osteoporosis can also be caused by disuse. Prolonged bone unloading, as seen in extended bed rest or space travel, inhibits bone formation and enhances bone resorption. This occurs due to the lack of appropriate regulation of bone mass by the osteocyte network and, possibly, through involvement of the sympathetic nervous system (13).

Although decreased BMD is what defines osteoporosis, this factor alone is not a major cause of pain or morbidity. Instead, morbidity associated with osteoporosis is primarily due to increased incidence of fragility fracture. Due to the biomechanical and biological alterations in osteoporotic bones, only a minor external force, such as a short fall, is required to induce a fracture. Osteoporotic fractures are three times more likely in women and typically occur after the age of 50 (14). Not only does osteoporosis increase fracture risk, but it is also associated with poorer fracture healing outcomes [reviewed in Cheung et al. (15)]. Current treatment strategies are aimed at increasing calcium and vitamin D levels through supplementation, inhibiting bone resorption through bisphosphonate administration, and mitigating the effects of menopause through hormone replacement (discussed in detail below). The FDA has also approved the anabolic agent, human parathyroid hormone (PTH) peptide, to treat osteoporosis. In addition, newer therapies, such as strontium ranelate administration and antibodies against RANKL, are being investigated (16). Stem cell therapies are being examined for their ability to enhance repair of fractures (17). Transplantation of allogeneic non-osteoporotic stem cells may be able to normalize the aberrant bone remodeling that occurs in osteoporotic patients, thereby reducing fracture risk (18). However, the optimal stem cell phenotype and method of delivery are still poorly characterized (19). There is an ongoing need to identify, develop, and improve therapeutics that reduce fracture risk, enhance bone mineral density, and promote fracture healing.

## Psychological Stress

Psychological stress is defined as an emotional experience that is accompanied by predictable biochemical, physiological, and behavioral changes (20). Psychological stress can occur in response to an acute event, as in a fight-or-flight response to a life-threatening or traumatic event, or stress can be chronic, as in the case of caregivers, service members, and high-stress occupations. In acute psychological and physical stress, stress signaling is initiated through the hypothalamic-pituitary-adrenal (HPA) axis and the sympathomedullary (SAM) pathway via secretion of stress hormones, which include glucocorticoids (cortisol) and catecholamines (epinephrine, norepinephrine). Immune cells (leukocytes) express receptors for these hormones (glucocorticoid receptors and adrenergic receptors, respectively) and rapidly respond to their induction by altering the inflammatory immune response. However, in chronic stress and chronic stress-associated mental health conditions, the HPA-axis becomes dysregulated, resulting in hypercortisolism or glucocorticoid resistance (21). Whether the stress response becomes pathologic is dependent on many factors, including individual coping skills, life history, severity, and duration of the stressor.

Anxiety or depression disorders can arise as a result of acute or chronic stress. Depressive mood disorders, such as major depressive disorder (MDD), are characterized by persistent emotional and physical symptoms, including depressed mood, loss of interest and enjoyment (anhedonia), and dysregulated sleep. Depression is often comorbid with anxiety, and both conditions can alter the HPA response. Anxiety can manifest as excessive worry, fear, irritability, difficulty concentrating, and with physical symptoms, such as increased heart rate and breathlessness. Mental health disorders, such as depression, result in a variety of potentially detrimental biochemical and physiological changes [reviewed in Yang et al. (22)]. Other stress-related conditions include acute stress disorder (ASD), in which individuals experience a constellation of symptoms, such as anxiety, flashbacks, and distress related to and surrounding a traumatic event. When these symptoms persist beyond the acute phase of 1 month, they are recognized as the chronic condition, termed post-traumatic stress disorder (PTSD). PTSD is recognized as an extreme case of chronic stress in which symptoms can persist for months to years. It is defined by display of symptoms that include heightened response to events or circumstances related to an initial traumatic and/or life-threatening event. Symptoms of PTSD are both intrusive, such as flashbacks and unwanted upsetting memories, and avoidant, such as evasion of stimuli that could recall the initiating trauma. Together, these symptoms have a significant impact on patient quality of life (QOL) and can lead to severe anxious, depressive, and debilitating effects (23). PTSD impacts approximately 3.6% of individuals annually, with increased incidence among Veteran populations, for whom rates have approached 20% in those returning from recent conflicts in Afghanistan and Iraq (24, 25). Estimates of the number of individuals with mental health disorders, or even those experiencing short-term psychological stress, are difficult to obtain, partially owing to the fear of stigmatism and rejection that may accompany mental health disorder diagnosis. Nonetheless, mental health disorders impact a significant percentage of the population, and it is becoming increasingly clear that psychological stress has significant impact on patient QOL, as well as physical health.

## Psychological Stress as a Risk Factor for Osteoporosis

Psychological stress can have lasting impact on risk for development of comorbid disease, as well as significant impact on pre-existing diseases. Chronic stress has been associated with obesity, atherosclerosis, lung pathologies, and diabetes (26, 27). In regard to osteoporosis, U.S. military veterans diagnosed with PTSD have a higher risk of developing osteoporosis (28), as do civilians with PTSD diagnosis (29). Likewise, it was found that among 73 female Holocaust survivors there was a 3.47-fold increase in prevalence of osteoporosis compared to controls (30), suggesting psychological stress may be a risk factor for osteoporotic disease. However, malnutrition and other factors likely played a role as well, although the authors did not discuss this possibility. A recent mouse study by Foertsch et al. showed that chronic stress induced by a chronic subordinate colony housing model of PTSD resulted in reduced growth plate endochondral ossification in adolescent mice (31). Increased expression of tyrosine hydroxylase (a catalytic enzyme involved in catecholamine biosynthesis) by bone marrow cells located in the growth plates of the femurs of chronically stressed mice suggested that decreased bone length and density may be due to stress-induced catecholamine impact on bone growth.

**Figure 1:**
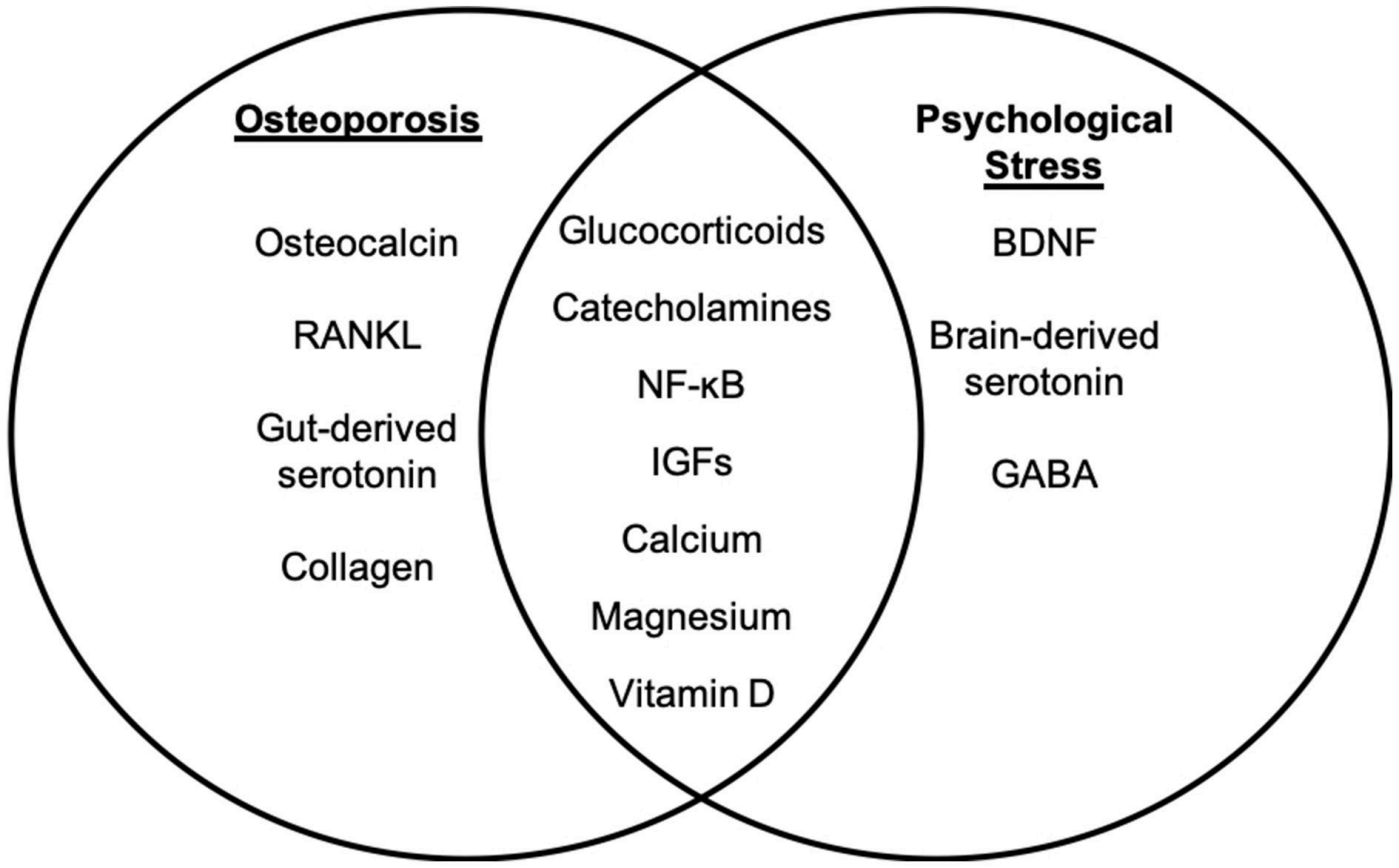
Molecular targets of osteoporosis and psychological stress. While psychological stress and osteoporosis occur via distinct mechanisms, there are several factors that overlap between psychological stress-associated mental health disorders and osteoporosis. The molecular factors that are distinct to osteoporosis **(left panel)** and psychological stress **(right panel)** are listed. Intersecting factors related to osteoporosis and psychological stress are listed in the **(middle panel)**.

**Figure 2:**
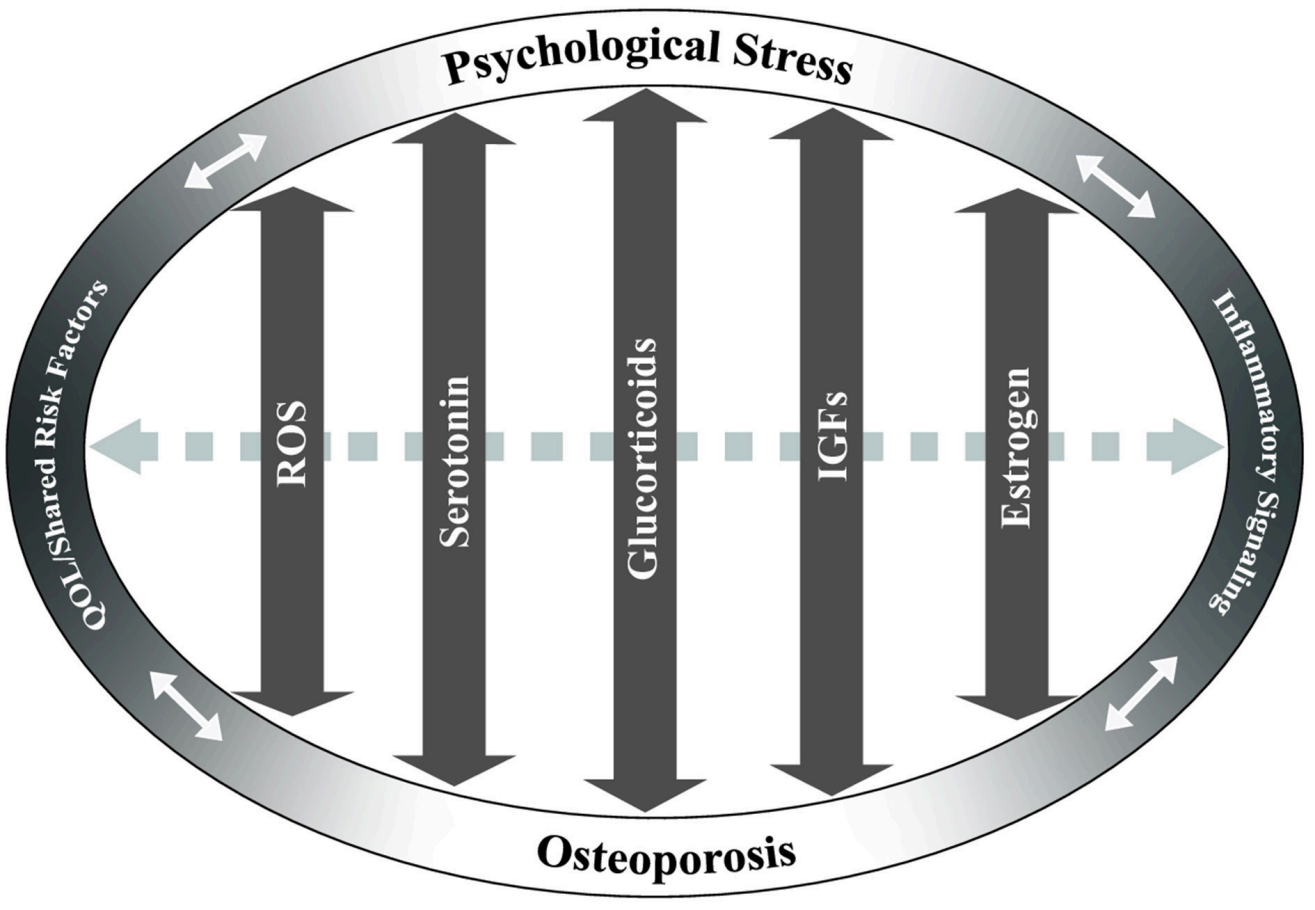
Potentially overlapping pathways of osteoporosis and psychological stress. Key pathways that may have overlapping effects between chronic stress and osteoporosis are identified based on current literature. These include reactive oxygen species (ROS), serotonin, glucocorticoid, insulin-like growth factor (IGF), and estrogen signaling pathways. Arrows indicate the potential of these pathways/factors to influence both chronic stress and osteoporosis in a bi-directional fashion. Dashed gray arrow between pathways indicates potential interactions between the pathways. Quality of life (QOL)/shared risk factors and inflammatory signaling are positioned at ends of ellipse to demonstrate cross-effects between chronic psychological stress and/or osteoporosis.

While the mechanisms underlying the physiological and biochemical impact of psychological stress on disease are not well-understood, several studies have shown that stress hormone signaling via the brain-immune connection is a significant contributor (32). Chronic stress has been associated with increased systemic inflammation (26, 27, 33) and altered hematopoiesis (34). Inflammatory factors have been shown to have a detrimental effect on osteoporosis through promotion of osteoclast differentiation and apoptosis of osteoblast populations [reviewed in Eastell et al. (35)]. It has also been suggested that a number of inflammatory factors may actually exhibit inhibitory effects on osteoclast activity, thereby potentially improving bone health in osteoporosis (36). Thus, while common to both conditions, the roles of inflammatory factors in osteoporosis and in psychological stress are likely highly complex and both context- and dose-dependent. A review of the current literature identifies several additional pathways and cellular mechanisms that are common to chronic psychological stress and osteoporosis. Literature is limited in terms of studies examining any direct mechanistic interaction between these pathways in the context of osteoporosis and psychological stress; however, independent examination of the mechanisms of disease and shared risk factors suggests that further research is warranted. The studies below, and summarized in Figures 1 and 2, discuss several of these common pathways, cellular and molecular mechanisms, and risk factors to highlight the potential for future examination of the role of chronic psychological stress/mental health on osteoporosis.

### Glucocorticoids

In chronic psychological stress, dysregulated glucocorticoid signaling has profound impacts on inflammation and may also contribute to disease risk (21). Stress-induced dysregulation of endogenous glucocorticoids may mimic the skeletal effects of glucocorticoid-induced osteoporosis. Glucocorticoids are hormones that exert their effects largely by entering the nucleus and modulating gene transcription. Glucocorticoid-responsive transcription factors are primary regulators of inflammation resulting from stress hormone signaling and include NF-κB. There is some evidence to suggest that activation of NF-κB through glucocorticoid responsive elements in response to psychological stress may contribute to the risk of osteoporosis through RANK signaling (37). In addition, glucocorticoids are known to act directly on bone cells, leading to decreased osteocyte viability, decreased osteoblast function due to reductions in IGF-2, and prolonged osteoclast viability [reviewed in Briot and Roux (12)]. Therefore, psychological stress may negatively impact bone health through modulation of endogenous glucocorticoids.

### Catecholamines

Catecholamines are stress hormones that include norepinephrine, epinephrine, and dopamine. Norepinephrine and epinephrine are released by the adrenal glands as part of the rapid fight-or-flight response to stress. This elevation is typically in response to a physical stressor; however, psychological stress (e.g., sudden bad news, fear, or PTSD-related flashbacks) can also trigger catecholamine release. Chronic and/or repeated elevations in norepinephrine or epinephrine in response to psychological stress may contribute to the development of depression (38). Dopamine is also increased in specific brain regions in response to pain or stress. Like other catecholamines, dopamine may become dysregulated in the case of chronic stress [reviewed in Vaessen et al. (39)].

One way in which psychological stress may impact osteoporotic disease risk and severity is through catecholamine-induced activation of β-adrenergic receptors on osteoblasts and osteoclasts. β-adrenergic receptor activation has been shown to increase RANKL expression, resulting in osteoclast differentiation (40). Treatment with a β-agonist resulted in bone loss due to increased bone resorption (41). β-adrenergic signaling was also shown to exacerbate bone loss through promotion of osteoclastogenesis via generation of reactive oxygen species (ROS) (42). These studies suggest that alterations in catecholamines due to chronic stress may impact bone health and contribute to risk and severity of osteoporosis.

### Myeloid Populations

Chronic psychological stress has been shown to alter myeloid phenotype and increase myelopoeisis. Activation and increased contribution of myeloid populations are significant in that myeloid-derived immune cells are the primary mediators of the inflammatory response promoted by chronic stress (43). The myeloid response to chronic stress may significantly contribute to bone health and osteoporotic disease, given that osteoclasts are myeloid-derived, and monocytes are well-known for their plasticity during wound repair. However, the role of myeloid cells in bone health is complex. Depletion of macrophages in mice was shown to lead to early skeletal growth retardation and osteoporosis and decreased the number of bone marrow-derived mesenchymal stromal cells (MSCs) present in the bones (44). Further, these so-called “osteomacs” were shown to be closely associated with areas of bone remodeling and were directly involved in formation of the canopy structure that makes up the bone-remodeling compartment. Depletion of osteomacs caused complete loss of this compartment. However, removal of osteomacs from calvarial cultures decreased markers of osteoblastic function, including osteocalcin (OCN) mRNA expression and mineralization *in vitro*. Thus, targeting the myeloid cell population as an osteoporotic treatment may not be an optimal approach due to the duality of its effects.

### Insulin-Like Growth Factors (IGFs)

Glucocorticoids and IGFs are known to regulate one another, suggesting that mood may influence levels of IGF-1 (45, 46). IGFs may also play a role in psychological stress (47) and osteoporosis (48). Circulating IGF-1 is increased in individuals with depression or anxiety disorders (49, 50) and has been shown to be a biomarker for vulnerability of an individual to stress following traumatic brain injury (51). Yu et al. found that, in a single prolonged stress model, IGF-1 levels were up-regulated by approximately 25% in the stressed group, although this data was not statistically significant (52). Another study by Hoshaw et al. demonstrated an anti-depressant and anti-anxiolytic effect of IGF due to its effects on serotonin (53). These conflicting reports suggests that further research is needed to determine whether IGF has beneficial or detrimental impacts on psychological stress-related mental health disorders.

In bone health, IGF-1 and−2 regulate osteoblast-osteoclast interactions, thus making them important regulators of bone remodeling (54). IGF-1 has also been shown to activate mammalian target of rapamycin (mTOR) remodeling to stimulate MSC differentiation into osteoblasts (55). Knockout of IGF-1 impairs osteoblast differentiation and leads to decreased trabecular bone formation (56). Its role in fracture healing is still not fully understood, as some studies suggest beneficial effects of IGF-1 treatment, while other studies demonstrate non-significant effects (57–59). IGF-1 action and circulating levels also decline with age, and this mechanism has been suggested to be an underlying cause of age-related osteoporosis [reviewed in Perrini et al. (60)]. IGF-2 is most commonly thought of as a fetal growth factor; however, it is the most abundant growth factor stored in adult bone. Induction of the osteogenic lineage from parthenogenetic embryonic stem cells is enhanced with IGF-2 treatment (61). Interestingly, different effects of IGF-1 vs. IGF-2 have been reported in human bone cell metabolic pathways, suggesting they activate different signaling cascades, even though they both primarily signal through the IGF1R (62, 63). The differing effects of IGF-1 and IGF-2 could be due to cell-specific expression patterns. It is also possible that the presence and concentration of specific insulin-like growth factor binding proteins (IGFBPs), which mediate IGF bioavailability and are temporally and spatially regulated, may regulate these differing effects. Together, these studies suggest IGF as a potential connecting pathway between osteoporosis and psychological stress. Additional studies are needed to delineate the role of IGF-1 vs. IGF-2 and to determine how IGFBPs (64) may be temporally and differentially regulated during osteoporosis, psychological stress, and in osteoporotic patients who have a history of mental health disorders.

### Oxidative Stress

Studies on depressive disorders have shown a significant decrease in neuronal and glial cells in depressed patients. It has been suggested that the decline in these populations is due to an increased amount of ROS [reviewed in Michel et al. (65)]. ROS have been shown to induce osteoblast apoptosis, leading to decreased bone formation (66, 67). ROS, as well as hydrogen peroxide (H_2_O_2_), are required for RANKL-induced osteoclast generation (68–70). Further, increased ROS in the bone marrow compartment can lead to expansion of lymphocytes, altered cytokine production (71, 72), and promotion of osteoclastogenesis (42). In regard to impacts on osteoporosis, ovariectomized rats were found to have increased oxidative stress compared to controls. However, treatment with palm tocotrienol, a potent antioxidant, for 8 weeks resulted in suppression of malondialdehyde levels, a marker of oxidative stress, and promotion of plasma glutathione peroxidase and erythrocyte superoxide dismutase activity, two key antioxidant enzymes (73). Thus, palm tocotrienols may have bone protective effects by limiting oxidative stress damage [reviewed in Chin and Ima-Nirwana (74)].

### Serotonin

Serotonin, or 5-hydroxytryptamine (5-HT), is a monoamine neurotransmitter that is involved in a host of important processes, including sleeping, eating, digesting, and mood regulation [reviewed in Sangkuhl et al. (75)]. Serotonin is synthesized both in the gut and in the brain by different isoforms of tryptophan hydroxylase (TPH), TPH-1 and TPH-2, respectively. The vast majority (95%) of serotonin is produced in the periphery, mainly by enterochromaffin cells in the duodenum. Until recently, it has been thought that serotonin does not interact with bone; however, recent studies have begun to unmask a complex role for serotonin in regulating bone mass and bone metabolism [reviewed in Wadhwa et al. (76)]. Serotonin has been shown to regulate osteoblast proliferation and function *in vitro* (77), and osteoblasts and osteoclasts express a variety of serotonin receptors (Htr1a, Htr1b, Htr1d, Htr2a, Htr2b) (78, 79). Addition of serotonin to RAW264.7 cells induced osteoclast differentiation through intracellular accumulation of serotonin via the serotonin transporter (SERT or 5-HTT), resulting in upregulation of NF-κB (80). When produced peripherally, serotonin inhibits bone formation and decreases osteoblast proliferation [reviewed in Ducy and Karsenty (81)]. When produced in the brain, serotonin acts as a neurotransmitter to exert a positive effect on bone mass accrual by enhancing bone formation and limiting bone resorption via regulation of the sympathetic response [reviewed in Dimitri and Rosen (82)].

### Shared Risk Factors

Several independent lifestyle risk factors for development of osteoporosis may also be impacted by concurrent stress-associated mental health disorders, such as smoking, alcohol use, and substance abuse. Smoking, in particular, represents a strong risk factor for development of osteoporosis. The direct mechanism(s) by which this occurs are not well understood. However, a study by Ko et al. demonstrated that serum from animals exposed to smoking resulted in increased osteoclast differentiation from macrophages in response to RANKL, as well as a reduction in alkaline phosphatase (ALP) and consequent reduction in osteoblast differentiation (83). In patients seeking mental health care, 28.2% report smoking, as compared to 17.5% among the general population (84). This finding suggests that psychological stress is associated with an increased risk for smoking. Due to the reported negative impact of smoking on bone health (85), psychological stress may also indirectly increase risk of osteoporosis. Similarly, alcohol consumption is a significant risk factor for development of osteoporosis (86, 87), due, in part, to senescence and ROS production in bone marrow-derived MSCs, which results in decreased osteogenic potential (88). Substance abuse, such as opioid addiction, is also elevated among those suffering with psychological stress-associated mental health disorders (18.7 vs. 5% among those without mental health disorders) (89). Increased rates of osteopenia and osteoporosis have been found among women addicted to opioids (90).

Obesity may represent another risk factor for osteoporosis, due to increased inactivity, leading to cases of unloading. Likewise, as described below, exercise may provide benefit for BMD and in reducing fracture risk. In addition, obesity leads to increased systemic inflammation, with many of the signals, such as NF-κB and TNF-α, being differentiation factors for osteoclasts as well. There is also a clear link between obesity and development of type 2 diabetes, which is another known risk fracture for development of osteoporotic fracture [reviewed in Walsh and Vilaca (91)]. However, weight gain, itself, may have positive effects on osteoporosis. Weight loss in postmenopausal women was shown to increase risk of frailty fractures (92). Conversely, weight gain reduced risk of hip fractures, although it does increase risk of other types of fracture (93, 94). Clearly, the effects of weight on fracture are complex and require further study.

Together, these studies indicate that, in patients with extreme and/or chronic psychological stress, osteoporotic risk may be exacerbated by compounded effects of these common risk factors. As such, in addition to independent risk factors for osteoporosis, the potential for a multifactorial feedback loop with psychological stress exists and should not be overlooked.

## Interaction of Treatments

Based on the studies above demonstrating potentially overlapping factors, cellular mechanisms, and signaling pathways between osteoporosis and chronic psychological stress, it is not surprising that treatments for these conditions may also have overlapping and opposing effects. Thus, it is critical that the interplay between stress and disease-mediated pathways is considered during the planning of best course of treatment for an osteoporotic patient, particularly one with a history of mental health disorder. While this review does not provide, and is not intended to provide, clinical recommendations, a discussion of current literature and potential crosstalk between treatments for osteoporosis and psychological stress-related mental health disorders is provided to encourage consideration of the implications of drug selection from a broad, whole-health perspective.

### Osteoporosis Treatments

Given the potential impact of psychological stress and its treatments on bone health, treatments that benefit both bone and mental health may be preferred, especially in patients at high-risk for concurrent osteoporosis and mental health disorders. In this section, we discuss common treatment options for osteoporosis, independent of type, and detail literature that provides evidence of potential impacts of these treatments on mental health. Literature findings outlined below are summarized in Table 1. For each osteoporotic treatment, we first discuss its primary use and impacts on bone health, followed by a review of current literature as to its effects on mental health.

**Table 1:** Interactions of treatments for osteoporosis and psychological stress.

**Drug/therapy**	**Relevant target**	**Osteoporosis**	**Psychological stress**	**Other considerations**
Bisphosphonates	Osteoclasts/bone mineral density	+	?	Potential improvement in mobility, which may improve QOL
Statins	TGFβ pathway	+	+/?	Cardiovascular impacts
Denosumab	RANKL	+	?	
Teriparatide	Parathyroid hormone	+	–/?	
Estrogen/SERM	Multiple	+	+/–	Long-term use could increase cancer risk
Strontium ranelate	Bone mineral density	+	?	
SSRI	Serotonin	-	+	
Benzodiazepines	GABA receptor	-	+	Cardiovascular impacts
Beta-blockers	β-adrenergic antagonist	+	+	Cardiovascular impacts
Barbiturates	GABA receptor	-	+	Addictive; No reversal agent
Fish oil (EPA and DHA)	Unknown/multiple	+	+/?	Cardiovascular impacts
Calcium	Bone mineral density	+	?	Kidney stone development
Magnesium	Bone mineral density	+	+/?	
Vitamin D	Required for calcium absorption	+	+/–	Kidney stone development
Exercise	Multiple	+	+	Multiple health benefits

*Treatments for osteoporosis and those for psychological stress potentially interact with each other. Positive benefit is denoted by “+”. Negative effects are denoted by “-”. Unknown or understudied effects are denoted by “?”. Relevant targets in context of osteoporosis or stress are listed. Considerations that may have important clinical impacts are included*.

It is worth noting that osteoporosis, particularly osteoporotic fracture, may affect mental health and QOL. Osteoporotic fractures can lead to poorer QOL outcomes and negatively impact physical, social, financial, and psychological well-being (95–97). In regard to psychological well-being, osteoporosis can lead to feelings of anxiety, due to fear of falling or fear of fracture, and depression. It has been shown that anxiety and depression are comorbidities of osteoporosis (98), and osteoporotic fracture can cause reduced self-esteem and self-image, likely due to feelings of helplessness and loss of independence (99). All of these factors (loneliness, anxiety, depression, loss of independence, reduced self-esteem, loss of social role, etc.) may, in turn, contribute to disease exacerbation. Effective management of osteoporosis that reduces incidence of osteoporotic fracture (e.g., effective caregiver support) likely provides substantial indirect mental health benefit by preventing these negative outcomes. As described below, many treatments for osteoporosis may also have direct, biochemical effects on mental health.

#### Bisphosphonates

Bisphosphonates are antiresorptive agents that bind to hydroxyapatite crystals and become ingested by osteoclasts, where they suppress an enzyme involved in osteoclast-mediated bone resorption. This slows the rate of bone remodeling. In addition, they have been well-documented to reduce fracture risk [reviewed in Crandall et al. (100)]. However, bisphosphonates have negative effects on fracture repair, as they interfere with maturation of cartilaginous callus to mature bone (101). Furthermore, atypical femoral fractures and osteonecrosis of the jaw are two serious side effects of extended bisphosphonate use [reviewed in Black and Rosen (102)]. While bisphosphonates are effective at stalling bone loss, they cannot restore bone mass, as they are antiresorptive and not anabolic. Thus, they may be less effective for patients presenting with severe bone loss.

Bisphosphonates are often the first-choice treatment for osteoporosis, however, there are a limited number of studies, to date, that have examined potential implications of bisphosphonate treatment on mental health. Citraro et al. demonstrated that treatment of ovariectomized rats, a model of osteoporosis, with sodium alendronate had short-term benefit on anxiety and had beneficial impacts on motor performance (103). Reduced immobility, increased distance traveled, and increased mean velocity in behavioral assessments were shown in ovariectomized rats following 3 months of sodium alendronate treatment. However, benefit was not maintained following 6 months of treatment, and short-term benefit can likely be attributed to improved mobility. This suggests that bisphosphonate treatment may provide positive impact on QOL outcomes, due to improved mobility, and may have short-term benefit for anxiety and depression, particularly for patients with type I osteoporosis. An important consideration with bisphosphonate treatment, however, is non-compliance due to incidence of flu-like illness and gastrointestinal upset associated with their use, as well as complicated dosing schedules (104). Non-compliance may be further increased among patients with PTSD, therefore, additional follow-up may be necessary (105). Studies by Kastelan et al. have demonstrated that a monthly, rather than weekly, dosing schedule may also be beneficial toward improving compliance and QOL (106).

#### Denosumab

Denosumab is a monoclonal antibody to RANKL, a ligand expressed by osteoblasts that is necessary for the differentiation of osteoclasts. Denosumab sequesters RANKL and prevents its interaction with osteoclastic RANK, mimicking the natural function of osteoprotegerin (OPG). The resulting decrease in osteoclastogenesis is associated with significant increases in BMD, which have been shown to continue for up to 10 years of treatment (107). Treatment with denosumab also decreases risk of hip, vertebral, and non-vertebral fractures (108). However, cessation of denosumab leads to a rapid rebound in bone turnover, which has raised concerns over multiple vertebral fractures (109, 110). Denosumab is administered as a subcutaneous injection every 6 months, which has been associated with higher compliance and greater patient satisfaction (111).

The effect of denosumab treatment on mental health is currently unknown. However, Suzuki et al. demonstrated that denosumab treatment altered levels of serum bone-related minerals in osteoporotic patients with rheumatoid arthritis, including alteration of magnesium levels, which is known to impact mental health (described below) (112). Further, recent reports are expanding our understanding of the role of the RANKL-RANK axis outside of the skeletal system [reviewed in Nagy and Penninger (113)]. RANKL is also expressed by T cells, which is thought to underlie the decrease in BMD associated with diseases of chronic T cell activation (114). RANKL-expressing T cells are known to home to the CNS, where they interact with RANK-expressing astrocytes and microglia (115, 116), cell types with an increasingly apparent role in the central response to chronic stress [reviewed in Calcia et al. (117)]. How denosumab affects immunomodulation of the CNS by RANKL-expressing T cells remains to be elucidated. There is also some evidence to suggest that activation of the sympathetic nervous system, commonly associated with chronic psychological stress, affects the peripheral expression of RANKL on osteoblasts (118, 119) and T cells (120). However, it is currently unknown how altered RANKL expression modulates the efficacy of denosumab in individuals with chronic psychological stress.

#### Estrogen Replacement Therapy/Selective Estrogen Receptor Modulators (SERMs)

Estrogen replacement can prevent postmenopausal bone loss and reduce fracture risk (121–125). The lack of estrogen in postmenopausal women causes dysregulation of bone cell differentiation, alters osteoblast/osteoclast activity, and induces osteoblast and osteocyte apoptosis, thereby leading to increased bone turnover, with a net effect of resorption exceeding formation (126–128). This is due to increased secretion of pro-inflammatory factors, such as IL-1 IL-6, and TNF-α, as well as estrogen's regulatory role in osteoclast receptor signaling, including RANKL and OPG (129–133). In addition, estrogen loss leads to decreased production of IGF-1, transforming growth factor β (TGFβ), and COL1, all of which are involved in stimulating osteoblast differentiation and activity. Estrogen replacement therapy, in effect, reverses these changes (134–137). However, the anabolic effects of estrogen on bone are dependent on preparation, dose, and route of administration (138). Likewise, estrogen plays a significant role in many tissues throughout the body, so systemic replacement of estrogen creates a complex clinical scenario. For example, a study by the Women's Health Initiative found that estrogen replacement had beneficial effects on fracture and colon cancer risk, but also increased incidence of cardiovascular events, strokes, pulmonary embolisms, and invasive breast cancers (139). Thus, although clearly effective, these systemic effects have lessened enthusiasm for estrogen replacement therapy as the first-line treatment option for osteoporosis (140).

Similarly, selective estrogen receptor modulators (SERMs) have been studied for their impacts on BMD and fracture risk. SERMs are compounds that interact with estrogen receptors and, like estrogen replacement, have broad systemic effects. Raloxifene has been widely used for treating osteoporosis, although its effects on BMD are modest, and it appears to only impact vertebral fractures (141, 142). Long-term use of raloxifene also decreased breast cancer risk, but increased risk of thromboembolic events (108, 143–145). Thus, as with estrogen replacement, SERMs are unlikely to be the gold standard treatment option for osteoporosis, but may be particularly beneficial for women with a strong family history of estrogen receptor-positive invasive breast cancer (146).

Along with impacting a range of tissues, estrogen has profound effects on mental health and is a known regulator of the stress response (147–149). Postmenopausal estradiol therapy provides protective effects against stress-induced cognitive effects, particularly working memory (150). Estrogen also positively impacts distribution of serotonin receptors, suggesting a role for estrogen in mood regulation (151, 152). Glover et al. found that low circulating estrogen levels are associated with higher fear-potentiated startle and fear extinction deficits in women with PTSD (153). Therefore, low levels of estrogen may play a role in PTSD by decreasing fear inhibition. Though several studies have demonstrated a positive impact of estrogen on mental health, negative effects of estrogen on brain activity and memory formation have also been observed. Shansky et al. showed that estrogen treatment impacted activity in the medial prefrontal cortex, resulting in increased sensitivity to working-memory impairment caused by pharmacologic and restraint stressors, possibly through regulation of the alpha-2a adrenergic receptor (154). Dysfunction of the medial prefrontal cortex is associated with stress-related disorders, including major depressive disorder and PTSD. Estrogen may also lead to increased intrusive memories, thereby influencing memory of emotionally arousing events (155). Further, estrogen has been shown to play a role in the pathophysiology of migraines, which are linked to depression, anxiety, abuse, and PTSD (156). Thus, estrogen plays a major and complex role in the stress response and may have both positive and negative effects on different areas of the brain. Estrogen's effects on cognition are likely further complicated by age and hormone status of the patient. It is of note that raloxifene does not appear to affect memory or cognition, and, thus, may be a better treatment option than estrogen replacement for women with a history of mental health disorders (145).

#### Statins

Statins have been prescribed for the treatment of cardiovascular disease for decades, but are just beginning to be investigated for their anabolic impact on bone [reviewed in Ruan et al. (157)]. Statins have been shown to influence bone remodeling through the BMP pathway and may inhibit osteoclast differentiation through increased BMP-2 expression (158). Statins may also regulate the RANK pathway to inhibit osteoclastogenesis. Bone formation may be promoted by statins through inhibition of osteoblast apoptosis mediated by Smad3 deletion via the TGFβ pathway. Currently, simvastatin and atorvastatin have been shown to have clinical efficacy (increased BMD) in the treatment of osteoporosis, while trials with other statins, such as pravastatin and rosuvastatin, failed to meet study primary outcomes of reduced fracture risk [reviewed in Wang et al. (159)]. Statin bioavailability in bone is low and may explain the lack of full efficacy despite the strong role in bone anabolism demonstrated in animal models and basic laboratory studies (160, 161). However, additional studies and efforts to improve bioavailability should be pursued as a result of the promising outcomes of early clinical and basic studies.

Statins may have beneficial effects on depression and anxiety. Simvastatin had an anti-depressant effect in a chronic mild stress model (162). However, data is conflicting with respect to patient benefit (163, 164). Epidemiological data suggests a potential positive effect, especially as an adjunctive therapy [reviewed in Salagre et al. (165)]. Statins also have anti-inflammatory and anti-oxidant functions, which may be beneficial for both stress-related pathologies and osteoporosis [reviewed in Bedi et al. (166)]. However, their use for treating psychological stress or osteoporosis concurrent with psychological stress requires additional examination.

#### Strontium Ranelate

Strontium ranelate is a divalent cation, similar to calcium, and can be administered daily in powder form to treat osteoporosis. Although its effects are weak, it is approved in Europe for treatment of osteoporosis in postmenopausal women and in men at high risk of vertebral and hip fractures who cannot use other pharmacological agents, such as bisphosphonates (16). It results in large increases in BMD, but this is partially due to the heavier strontium ions physically replacing calcium ions within the hydroxyapatite. In postmenopausal women with established osteoporosis, 4-year treatment with strontium ranelate reduced incidence of vertebral fractures by ~40% and non-vertebral fractures by 16%, while hip fractures were found to be reduced only after *post-hoc* analysis of a high-risk patient subgroup (167, 168). While a potentially effective antiresorptive agent, strontium ranelate may be associated with increased risk of cardiovascular events and, thus, patients must be closely monitored (169).

There are minimal studies that have examined the direct effect of strontium ranelate on mental health. However, improved QOL outcomes associated with its use may offer benefit to those experiencing psychological stress. For example, oral administration of strontium ranelate in postmenopausal women with established vertebral osteoporosis resulted in prevention of QOL impairment compared to placebo group, with clear improvement in emotional and physical dimension scores (170). In addition, a 2008 multicenter trial in Russia analyzed the effect of 1-year administration of strontium ranelate on BMD of patients with postmenopausal osteoporosis. It was found that strontium ranelate increased lumbar vertebra BMD, inhibited local tissue-mediated bone resorption markers, and resulted in improved QOL outcomes, with patients reporting better motility, lowered rates of depression, and improved self-assessments (171). While promising, further studies are needed to determine the impacts of strontium ranelate on mental health and whether or not it may serve as a more suitable treatment option for osteoporotic patients with a history of mental health disorder.

#### Teriparatide

Teriparatide is a recombinant form of PTH, consisting of the bioactive N-terminal 34 amino acids. PTH is involved in regulation of serum calcium levels and is a stimulator of both bone formation and bone resorption. Teriparatide is the only FDA-approved anabolic bone agent for treating osteoporosis, but is, currently, cost-prohibitive (35, 172). Daily or weekly subcutaneous injections of teriparatide were shown to increase both spine and hip BMD (173). Neer et al. demonstrated that a 20 μg/daily dose of teriparatide resulted in ~70% reduction in vertebral fractures and ~50% reduction in non-vertebral fractures in women with low BMD and a previous history of vertebral fractures over a 21-month treatment period (174). However, teriparatide did not reduce hip fracture risk. Teriparatide is associated with a number of negative side effects, including nausea, headache, hypercalcemia, and musculoskeletal pain. In addition, benefits of teriparatide are quickly lost after discontinuation, and it is only approved for up to 2 years of use (102). Importantly, it has also been shown that co-therapy with teriparatide and alendronate does not provide advantage over monotherapy (175).

In regard to mental health effects, common symptoms of hyperparathyroidism overlap with those of psychological stress-associated mental health disease, including fatigue, anxiety, insomnia, and depression. The molecular, cellular, and biochemical mechanisms behind the relationship between PTH and mental health are not known. Recently, however, PTH levels were shown to negatively correlate with plasma corticosterone levels after acute restraint stress (176). In addition, a significant reduction in parathyroid hormone receptor 1 (PTHR1) levels in both the kidney and thyroid was observed in rats exposed to chronic (28-day) daily restraint stress. This potential link is supported by clinical data, which demonstrated that teriparatide resulted in increased plasma and urinary cortisol following sustained treatment (6 months-1 year) (177). These studies suggest clinical considerations should be made regarding the potential impact of teriparatide use on cortisol levels in osteoporotic patients with PTSD, depression, or anxiety.

### Psychological Stress Treatments

As described above, it is clear that osteoporosis, particularly osteoporotic fracture, and associated treatments may have substantial mental health impacts. Mental health disorders may also, in turn, have significant impact on bone health. Anxiety has been reported to contribute to lower hip BMD (178). Several studies have shown that depression is a predictive factor for osteoporosis and fracture development (179–181). In addition, pharmacological interventions targeted at improving mental health, particularly in patients with major depressive disorder or PTSD diagnoses, may impact bone health. In the subsections below, we describe commonly prescribed medications for PTSD and depression and then review current literature indicating impacts of these agents on bone health.

#### Selective Serotonin Reuptake Inhibitors (SSRIs)

Selective-serotonin reuptake inhibitors (SSRIs) have become a first-line treatment for patients with moderate to severe depressive disorders, as they are generally considered safe, well-tolerated, and associated with minimal severe side effects (182). SSRIs function by inhibiting serotonin (see section Serotonin) reuptake by the presynaptic neuron, thereby maintaining higher levels of serotonin in the synapse and increasing postsynaptic neurotransmission. As a result of this inhibition, there is a resultant increase in extracellular concentrations of serotonin in both the brain and periphery. In addition, SSRIs are most highly concentrated in the bone marrow, thus raising the concern as to their impacts on bone metabolism (183). Interestingly, SSRIs appear to exert a temporally regulated dual-effect on bone. Short-term SSRI administration results in elevated systemic serotonin levels, but these levels are reduced by about 50% over a longer treatment period [reviewed in Ducy and Karsenty G (81)]. In a rigorous study by Ortuno et al., fluoxetine was shown to act on bone remodeling via two distinct mechanisms in mice. When used for <3 weeks, fluoxetine acts peripherally to cause anti-resorptive effects by directly impairing osteoclast differentiation and function through a serotonin-reuptake-independent mechanism that is dependent on intracellular Ca^2+^ levels and the transcription factor, Nfatc1. In addition, these effects were reversible, thus ruling out cell death as the reason for the observed anti-resorptive effects. No effect of fluoxetine was observed on osteoblasts. However, when fluoxetine was given to mice for 6 weeks, it triggered a brain serotonin-dependent rise in sympathetic output that increased bone resorption sufficiently to counteract its local anti-resorptive effect, which led to a net effect of impaired bone formation and bone loss. Hypothalamic extracts from mice treated for 6 weeks with fluoxetine showed significantly lower levels of p-CREB, a downstream mediator of serotonin signaling through Htr2c. The study also found that it was possible to neutralize this long-term effect of fluoxetine treatment through co-treatment with the beta-blocker, propranolol, which leaves the localized peripheral effect intact and prevents fluoxetine-induced bone loss (184).

In accordance with these findings, clinical studies illustrate that, with chronic usage of commonly prescribed SSRIs, bone health is negatively impacted. In numerous studies, SSRIs have been found to increase risk for secondary osteoporosis, lower BMD, and increase incidence of both hip and vertebral fractures (185–192). The direct mechanisms by which SSRIs impact bone health, particularly in humans, are still not wholly understood, particularly due to temporal and location-specific effects of serotonin. However, a promising potential treatment option may be LP533401, an inhibitor of Tph1, that does not cross the blood-brain barrier, thus will not affect synthesis of brain serotonin. LP533401 has been shown in a Phase II clinical trial to not exhibit significant toxicity or side effects in patients being treated for irritable bowel syndrome (193). In rats, LP533401 administration once daily by oral gavage for up to 6 weeks resulted in full rescue of osteoporosis in ovariectomized rodents in a dose-dependent manner (194, 195). Taken together, these studies would suggest that it is important to consider a patient's history of SSRI use when treating osteoporosis, as any benefit received from an osteoporotic drug, such as a bisphosphonate, could be countered by concurrent SSRI use [reviewed in Haney et al. (196)].

#### Benzodiazepines (anxiolytics)

Benzodiazepines are another commonly prescribed treatment for psychological stress, especially as a second-line or adjunctive medication. These drugs enhance the signaling of the neurotransmitter, gamma-Aminobutyric acid (GABA), through GABA receptors, and may also increase dopamine signaling to reduce anxiety that is often associated with stress. Benzodiazepines are central nervous system depressants and, thus, have sedating effects.

Benzodiazepines have been shown to have significant negative impact on bone health, primarily due to increased fall risk (197). Benzodiazepines have also been shown to decrease osteoblast differentiation through benzodiazepine-like receptors. BMD may also be reduced as a result of benzodiazepine treatment, and their use has been associated with increased ALP, reduced serum levels of vitamin D [reviewed in Fan et al. (198)], and increased levels of prolactin, which, in turn, results in decreased estrogen (199). These studies strongly suggest that benzodiazepines be prescribed with caution among those at risk for development of osteoporosis, and lifestyle modifications and supplementation as adjunctive therapies warrant consideration in these patients.

#### Barbiturates

Barbiturates, derived from barbituric acid, are a class of central nervous system depressants and are classified as anti-epileptic drugs. Barbiturates are GABA receptor agonists, exerting their effect by blocking transmembrane receptors for the primary excitatory neurotransmitter in the central nervous system, glutamate. The resulting activation of inhibitory GABA signaling coupled with inhibition of excitatory neurotransmitters causes sedation. These drugs are highly addictive and do not have a reversal agent in the case of overdose. Therefore, barbiturates are not as widely prescribed today as they have been in the past. However, these drugs are still prescribed for treatment of anxiety, seizures, migraine headaches, and in the elderly as sleep aids.

Barbiturates are detrimental to bone due to impacts on calcium and vitamin D metabolism and absorption. Although the negative effects of barbiturates are likely multifactorial and complex (200), perhaps the most recognized mechanism is through increased cytochrome p-450 enzymatic activity, which results in production of an inactive form of vitamin D, thereby leading to a reduction in calcium absorption from the gastrointestinal tract. Reduced vitamin D and calcium levels stimulate production of PTH and perpetuate bone loss due to calcium resorption from bone (201, 202). Due to their significant effects on bone resorption, use of barbiturates has been noted as a cause of secondary osteoporosis. As with other CNS depressants, barbiturate use is associated with increased risk of fracture due to an increased fall risk resulting from gait disturbances. Thus, due both to the risk of addiction and implication in osteoporosis, as well as elevated fracture and fall risk, alternatives to barbiturates for the treatment of psychological stress may be preferred.

#### Beta-Blockers

Beta-blockers act to inhibit β-adrenergic signaling and are commonly prescribed for the treatment of hypertension. More recently, beta-blockers, such as propranolol, have been prescribed for other conditions, including anxiety. The use of beta-blockers for PTSD has also been suggested, with the goal of preventing detrimental memory relapse of traumatic events [reviewed in Roque (203), Burbiel (204)]. However, due to concerns regarding potential negative impact on depression, these medications are not necessarily considered front-line treatments for psychological stress-associated mental health disease.

Given the impact of catecholamines on bone health (discussed above), it is not surprising that beta-blockers may have beneficial impacts on osteoporosis (205) and have been shown to reduce fracture risk by as much as 50%. In a study of men over the age of 55, long-term (> 5 years) beta-blocker use was associated with increased maxillary BMD compared to those on calcium channel blockers for hypertension (206). In a U.K. study, beta-blocker treatment was associated with reduced fracture risk (207). This was also demonstrated in an Australian study, in which women on beta-blockers were shown to have decreased fracture risk and increased BMD of the hip and ultradistal forearm (208). *In vitro* studies suggest that the positive impact of beta-blockers on bone health may be due to promotion of bone formation by osteoblasts, increased osteoblast numbers, decreased osteoclast numbers, and impairment of osteoclast-mediated bone resorption (209).

## Lifestyle Modification and Dietary Supplements

In this section, we provide a perspective outlook on lifestyle modifications and dietary supplements that may have beneficial effects on bone health (210) and may reduce psychological stress (211). For example, it has been suggested that the Mediterranean diet may have positive impacts on bone health, whereas the modern Western diet causes a state of low-grade chronic inflammation that promotes osteoporosis (212–214). A movement toward complimentary, alternative and integrative medicine has provided insight into the benefits of adjunctive and naturopathic remedies. Calcium and vitamin D supplementation have well-recognized benefits toward improved bone health and reducing osteopenia. However, their impacts on psychological stress are less well-studied. Other alternative therapies that have been gaining attention include magnesium supplementation and fish oil/omega-3 supplementation. The benefits of exercise in promoting overall health are well-recognized. Recent studies regarding several lifestyle modifications and dietary supplements and their effects on bone health and psychological stress are described below. These alternatives may offer complementary benefit with reduced risk compared to traditional pharmacological intervention, and, therefore, warrant additional study toward potential impact on patient outcome for those with, or at high risk for, osteoporosis and concurrent psychological stress-associated mental health disorders. In the subsections below, literature supporting alternative or adjunctive therapies are discussed. First, the literature indicating effects on bone health are described, followed by a review of the literature indicating impact on mental health.

### Exercise

Besides obvious beneficial effects on muscle mass, weight-bearing and resistance exercises can lead to increases in BMD (215). Although this impact may be more beneficial at a young age, some studies have shown that exercise increases BMD in postmenopausal women (216, 217). Longitudinal studies using high-resolution computed tomography scans have shown that regular physical activity improves skeletal microarchitecture (218). Further, exercise and balance can limit fall risk (102). The converse is also true, in that low levels of physical activity are associated with bone loss and >2-fold risk of fracture (219, 220). However, robust data is still lacking on whether there are any beneficial effects of long-term exercise on fracture susceptibility.

It is well-established that regular exercise can improve mental health. Participation in exercise programs has been shown to improve symptoms in patients with anxiety-, stress-, and trauma-related disorders, with positive effects lasting beyond the scope of the training program (221–224). In some cases, exercise therapy was more effective in reducing anxiety than traditional forms of therapy, including psychotherapy and pharmacotherapy (222, 225, 226). The benefits of short- and long-term aerobic exercise on overall mental health and function are multifold. On a biochemical level, exercise has been shown to reverse some of the neurological changes induced by exposure to psychosocial and/or physical stressors, including release of hippocampal corticosterone, decreased neurogenesis, and impaired hippocampal-dependent behaviors, such as learning and memory (227–230). In animal models of stress, both forced and voluntary exercise interventions have been shown to restore neuronal differentiation in the hippocampus (231, 232), increase levels of hippocampal brain-derived neurotrophic factor (BDNF) (233–235), and restore cognitive function (233, 236). There is also evidence that exercise-induced neurochemical changes, including increased production of hippocampal BDNF and altered hippocampal glucocorticoid receptor levels, may be protective against the stress response (237–239). On a psychological level, exercise may act as an interoceptive exposure (240, 241), in which patients with PTSD and anxiety-related disorders are sensitized to feared somatic sensations (242, 243). Alternatively, exercise may produce its anxiolytic effect by offering a distraction from distressing thoughts (244, 245) and/or inducing neurochemical changes, such as increased endorphin production (246). The biochemical mechanisms by which exercise alleviates symptoms in patients with anxiety and PTSD have not been fully elucidated and require further investigation.

### Calcium and Vitamin D

Nutrition and intake of appropriate levels of vitamins and minerals play a key role in maintenance of a healthy skeleton. In regard to osteoporosis, calcium and vitamin D supplementation have been the most studied to date. The Women's Health Initiative conducted a large randomized trial involving more than 36,000 postmenopausal women to determine the efficacy of 1,000 mg of calcium combined with 400 IU of vitamin D supplementation daily. It was found that this combination did not significantly impact risk of hip fracture, although *post-hoc* analysis demonstrated benefits for women age 60 years of age or older and those who adhered most strictly to the treatment schedule (247). In contrast, a 2016 meta-analysis of randomized controlled trials found a significant 15% reduced risk of total fractures and a 30% reduced risk of hip fractures with calcium and vitamin D supplementation (248). However, there has been no evidence, to date, that vitamin D supplementation alone reduces fracture risk, although it may reduce fall risk (249). Effects of supplemental calcium alone on fracture risk are still unknown, as no large-scale, randomized trials have been conducted (102). In addition, vitamin D and calcium supplementation were, not surprisingly, shown to increase risk of kidney stone development by 17% (247). Thus, at this time, vitamin D and/or calcium supplementation alone is not considered an appropriate treatment for osteoporosis.

In regard to mental health, several studies have examined the effects of vitamin D and calcium. Vitamin D is known to play a role in depression (250–252), and vitamin D receptors are present in multiple brain regions (253). Further, recent studies have begun to demonstrate a relationship between anxiety and serum levels of vitamin D (254). This may impact quality of life, particularly in postmenopausal women at increased risk of osteoporosis (255, 256). Vitamin D has also been shown to increase synthesis of neurotransmitters, including dopamine and norepinephrine, in rats (257). However, in a large-scale randomized, double-blinded US trial, no relationship was found between vitamin D/calcium supplementation and depression in over 36,000 postmenopausal women (258). In contrast, a Norwegian randomized, double-blind controlled trial found that weekly administration of vitamin D for 1 year in normal, healthy adults resulted in improved scores for depression compared to placebo (259). In a Korean study, low-dietary calcium was found to be associated with increased depression in middle-aged women (260). In women with premenstrual syndrome, supplementation with 500 mg of calcium carbonate twice daily for 3 months resulted in improvements in parameters assessing early tiredness, appetite changes, and depressive symptoms (261). There are also studies to suggest that calcium supplementation can be used to mitigate symptoms of postpartum depression (262). Thus, there is accumulating evidence of the beneficial effects of vitamin D and/or calcium supplementation on depression.

### Magnesium

Magnesium is the fourth most abundant cation in the body and is involved in cardiovascular, bone, and brain health, as well as maintenance of homeostasis (263, 264). Supplementation with magnesium is generally well-tolerated with limited side effects. For bone, magnesium supplementation has been less well studied than calcium and/or vitamin D. However, bones store approximately 60% of total body magnesium, and its release is dependent upon bone resorption (265). In rats, it has been shown that decreased dietary magnesium leads to a reduction in vitamin D, ALP, and OCN levels, as well as decreased bone volume and trabecular thickness (266). Tucker et al. demonstrated that magnesium intake was associated with increased BMD at one hip site for men and women and in the forearms of men (267). In a 2014 Women's Health Initiative study, it was found that postmenopausal women who consumed >422.5 mg of magnesium had slightly higher (2–3%) BMD than women who consumed <206.5 mg of magnesium daily. In addition, magnesium consumption correlated with increased physical activity, but also increased fall risk (268). A 2017 study demonstrated that dietary magnesium intake led to a 27% decrease in fracture risk (269). Thus, maintaining appropriate levels of magnesium appears to be beneficial in the maintenance of bone integrity.

Magnesium supplementation has been suggested for its anxiolytic effects and has shown promising results in clinical studies. However, additional examination is required to develop appropriate treatment recommendations [reviewed in Boyle et al. (270)]. Low magnesium intake (271) and low serum levels of magnesium have been associated with depression [reviewed in You et al. (272)]. Several studies have also demonstrated positive effects of magnesium supplementation for depression (273, 274). Magnesium has also been used to improve sleep, especially among those with magnesium deficiency. Mechanistic studies of magnesium supplementation on depression have been limited. However, in a model of chronic mild stress, it was demonstrated that magnesium may exert its anxiolytic and anti-depressive effects in part by acting as a GABA agonist and as an inhibitor of N-methy-D-aspartate receptor (NMDAR) (275). Based on these positive effects on mental health and bone integrity, as well as limited negative side effects, magnesium supplementation may serve as a beneficial supplement for osteoporotic patients with a history of mental health disorder.

### Omega-3 Fatty Acids

Polyunsaturated fatty acids, including eicosapantaenoic acid (EPA) and docosahexaenoic acid (DHA), are commonly contained in fish oil supplements and fatty fish, such as salmon, tuna, mackerel, and sardines. The ratio of these fatty acids varies across fish oil supplements and can have significant impact on effect and balance of these omega-3 and omega-6 fatty acids in the body. This balance is critical toward the beneficial anti-inflammatory effects of fatty acid supplementation. Due to these anti-inflammatory effects, there have been a handful of studies examining the impacts of fatty acids on bone health [reviewed in El-Sayed and Ibrahim (276)]. Using bone marrow-derived macrophages, Kim et al. demonstrated that DHA led to suppression of macrophage colony-stimulating factor (M-CSF)-induced proliferation of osteoclast precursors. This effect was likely mediated through decreased Akt activation and downregulated cyclin D1 and D2 expression. In addition, DHA led to increased apoptosis in mature osteoclasts (277). In rats, a diet supplemented with chia seeds, which are fatty acid-rich, was shown to increase BMD in the tibia (278). Lavado-Garcia et al. observed a similar effect, with long-chain omega-3 polyunsaturated fatty acid intake contributing to an increase in BMDs in the hips and lumbar spine of normal and osteopenic, but not osteoporotic, Spanish women (279). In a randomized, double-blind, placebo-controlled trial, Dong et al. reported that omega-3 polyunsaturated fatty acid supplementation led to decreased bone turnover by decreasing serum levels of bone-specific ALP and OCN over time (280). However, it was stated that higher doses and a longer duration needed to be tested before a definitive statement could be made as to the effects of fatty acids on bone metabolism. In a systemic review and meta-analysis, Shen et al. suggest that the primary impact of omega-3 fatty acids on bone is a reduction in serum OCN (281). However, there is still a lack in mechanistic understanding of how omega-3 fatty acids may be mediating these effects on bone, particularly since even just one fatty acid can trigger multiple independent pathways (282).

Depression and anxiety have been associated with reduced levels of polyunsaturated fatty acids (283, 284). Accordingly, several studies have demonstrated a positive effect of fatty acid supplementation [reviewed in Burhani and Rasenick (285)]. One study in rats comparing the effects of EPA and DHA supplementation demonstrated increased anxiolytic effects of EPA (286). Another study demonstrated anti-depressant effects of fish oil supplementation in rats subjected to chronic unpredictable mild stress (287). Fish oil supplementation may also improve the physiological symptoms of psychological stress. Fish oil supplementation has been shown to reduce the effects of mental stress (serial subtraction exercises) on heart rate, calf vascular conductance, and muscle sympathetic nerve activity (288). In contrast, a more recent study demonstrated no benefit of EPA supplementation on perceived psychological stress (289). Therefore, while studies have been promising regarding the use of omega-3 supplementation for treatment of psychological stress, including depression, anxiety, and PTSD, continued research is needed to determine the appropriate type of supplementation, dose, and application. Continued mechanistic studies are needed, but, to date, studies have suggested these supplements impart anti-inflammatory action and modification of neurotransmitter signaling through membrane and G-protein mechanisms [reviewed in Burhani and Rasenick (285)]. Overall, studies have shown promising benefit to multiple pathologies without significant negative impact. As such, fatty acid supplementation may warrant recommendation for concurrent osteoporosis and psychological stress.

## Conclusions

Together, the studies reviewed above suggest that, while osteoporosis and psychological stress occur via differing mechanisms, there are several potential molecular links that exist between a pathological response to stress and the development of bone disease. Although not a comprehensive list, these may include dysregulation of the HPA-axis and SAM pathway, inflammatory pathways, IGF signaling, estrogen, serotonin, GABA, and RANKL (Figures 1, 2). Consequently, an in-depth understanding of the mechanisms that regulate and intersect stress and bone health is needed to determine risk and treatment recommendations.

In addition, the pharmacological therapies used for mental health disorders and osteoporosis may have interacting effects (Table 1) that should be carefully considered in making treatment recommendations toward the most beneficial effect. These interactions are likely highly complex and influenced by a number of patient-specific risk factors, including lifestyle, genetics, epigenetics, and diet. Thus, there is a need for further basic and clinical research to determine the significance of chronic psychological stress on bone health. The multifactorial nature of diseases in treatment, lifestyle recommendations, in terms of making informed personalized medicine decisions should also be considered. Alternative or adjunctive therapies, such as lifestyle modification and dietary supplementation, may represent a novel approach to mitigating the effect of concurrent chronic psychological stress and osteoporosis, but further study is needed to examine the potential benefit of these options in this context. Overall, the interaction of psychological stress and osteoporosis is an important example of the need for additional research examining the broad, whole-health effects of chronic psychological stress on disease and the need for further study of the application of lifestyle modifications toward a personalized medicine approach.

## Author Contributions

RK and LM: conception and design, drafting, and revising of the manuscript. NJ, SS, and AL: drafting and revising of the manuscript. All authors read and approved the final manuscript.

### Conflict of Interest Statement

The authors declare that the research was conducted in the absence of any commercial or financial relationships that could be construed as a potential conflict of interest.

## Abbreviations

ALP: Alkaline Phosphatase
ASD: Acute Stress Disorder
BDNF: Brain-Derived Neurotrophic Factor
BMD: Bone Mineral Density
BMPs: Bone Morphogenic Proteins
CNS: Central Nervous System
COLI: Procollagen Type I
DHA: Docosahexaenoic Acid
EPA: Eicosapantaenoic Acid
FDA: Food and Drug Administration
GABA: Gamma-Aminobutyric Acid
GAD: Generalized Anxiety Disorder
H_2_O_2_: Hydrogen Peroxide
HPA: Hypothalamic-Pituitary Adrenal
IGF-1: Insulin Growth Factor-1
IGFBPs: Insulin Growth Factor Binding Proteins
mTOR: Mammalian Target of Rapamycin
MDD: Major Depressive Disorder
MSCs: Mesenchymal Stromal Cells
NFκB: Nuclear Factor Kappa B
NMDAR: N-methy-D-aspartate receptor
OCN: Osteocalcin
OPG: Osteoprotegerin
PTH: Parathyroid Hormone
PTHR1: Parathyroid Hormone Receptor-1
PTSD: Post-Traumatic Stress Disorder
QOL: Quality of Life
RANKL: Nuclear Factor Kappa B Receptor Ligand
ROS: Reactive Oxygen Species
SAM: Sympathomedullary
SERMs: Selective Estrogen Receptor Modulators
SSRI: Selective-Serotonin Reuptake Inhibitors
TGFβ: Transforming Growth Factor-β
TNF-α: Tumor Necrosis Factor-α.
